# ULBP2 Promotes Tumor Progression by Suppressing NKG2D-Mediated Anti-Tumor Immunity

**DOI:** 10.3390/ijms26072950

**Published:** 2025-03-24

**Authors:** Kohei Yamane, Kosuke Yamaguchi, Yasuhiko Teruya, Naomi Miyake, Yuji Nakayama, Takafumi Nonaka, Hiroki Chikumi, Akira Yamasaki

**Affiliations:** 1 Division of Respiratory Medicine and Rheumatology, Department of Multidisciplinary Internal Medicine, Faculty of Medicine, Tottori University, 36-1 Nishi-cho, Yonago 683-8504, Japan; yamanekohei1013@gmail.com (K.Y.); yteruya@tottori-u.ac.jp (Y.T.); otoyo.nao@gmail.com (N.M.); tnonaka@tottori-u.ac.jp (T.N.); yamasaki@tottori-u.ac.jp (A.Y.); 2 Division of Radioisotope Science, Research Initiative Center, Organization for Research Initiative and Promotion, Tottori University, 86 Nishi-cho, Yonago 683-8503, Japan; yujin@tottori-u.ac.jp; 3 Division of Infectious Diseases, School of Medicine, Faculty of Medicine, Tottori University, 36-1 Nishi-cho, Yonago 683-8504, Japan; chikumi@tottori-u.ac.jp

**Keywords:** ULBP2, NKG2D ligands, NKG2D, tumor immunology, immune evasion, NK cells, cancer immunotherapy

## Abstract

UL-16 binding protein 2 (ULBP2), a human NKG2D ligand, has been identified as a poor prognostic factor in several cancers based on recent comprehensive analyses of immune-related genes using the Cancer Genome Atlas datasets. Despite its clinical significance, the functional role of ULBP2 in vivo remains largely unknown. In this study, we investigated the role of ULBP2 in modulating anti-tumor immunity using murine melanoma cell lines engineered to stably express surface-expressed or soluble ULBP2. Subcutaneous transplantation of ULBP2-expressing melanoma cells into syngeneic mice resulted in accelerated tumor growth, mediated by surface-expressed ULBP2, through the suppression of NKG2D-dependent immune responses. In vitro experiments revealed that sustained exposure to tumor-expressed ULBP2 reduced NKG2D expression and cytotoxic activity of splenocytes. In contrast, soluble ULBP2 did not significantly affect tumor growth or immune responses. These findings suggest that surface-expressed ULBP2 plays a pivotal role in tumor immune evasion and highlight its potential as a therapeutic target to enhance anti-tumor immunity.

## 1. Introduction

The NKG2D receptor, an activating receptor of natural killer (NK) cells, is primarily expressed in NK and T cells, including NKT cells, CD4^+^ T cells, CD8^+^ T cells, and γδ T cells [1,2,3,4]. NKG2D ligands are frequently upregulated on cancer cells, bind to NKG2D receptors, and activate NK cells, leading to their degranulation and cytokine production, thereby promoting tumor cell destruction [5]. In humans, NKG2D ligands include UL-16 binding protein (ULBP) 1–6 and MHC class I chain-related proteins A and B (MICA and MICB). However, in mice, NKG2D ligands include Rae1α-ϵ, H60a-c, and MULT-1 [1,6,7,8].

The role of NKG2D ligands in anti-tumor immunity is profoundly complex. Ectopic expression of murine NKG2D ligands on tumor cells enhances tumor immune elimination [9,10]. However, chronic stimulation of NKG2D by murine NKG2D ligands promotes tumor progression [11,12]. Regarding human NKG2D ligands, in vitro studies have reported that tumor cells expressing MICA, MICB, or ULBP2 are more susceptible to NK cell-mediated cytotoxicity [1,13,14,15,16]. In contrast, the soluble forms of these ligands shed from the tumor cell surface, downregulate NKG2D expression, and suppress anti-tumor immunity [17,18]. Thus, the effects of NKG2D ligands on anti-tumor immunity vary depending on whether immune cells are exposed to them acutely or chronically and whether they are surface-expressed or in soluble form [12].

ULBP2 is associated with poor prognosis in patients with cancer based on clinical data. An elevation of soluble ULBP2 in the serum has been reported as a poor prognostic factor in patients with non-small cell lung cancer [19], melanoma [20], and pancreatic cancer [21,22]. Furthermore, recent comprehensive analyses of immune-related genes using the Cancer Genome Atlas dataset revealed that high ULBP2 expression in tumor tissues is one of the top poor prognostic factors in colorectal [23,24,25,26], head and neck [27,28], and breast [29,30] cancers. Similarly, analyses of NanoString PanCancer Immune Profiling data from multiple studies covering ten cancer types revealed ULBP2 as part of an immune gene signature associated with shorter overall survival in both solid and blood malignancies [31]. These clinical findings indicate that ULBP2 may promote tumor progression and could represent a potential target for cancer immunotherapy.

Despite extensive clinical data suggesting its significance, the specific impact of ULBP2 in tumor-bearing animal models with fully competent immune systems remains unclear. While murine tumor cells stably expressing ULBP2 are reportedly rejected upon transplantation into syngeneic mice [14], the functional role of ULBP2 expressed by tumor cells in transplanted and established tumors in modulating anti-tumor immunity has not been thoroughly investigated.

Therefore, to bridge this gap, we generated murine melanoma cell lines (B16F10 and B16BL6) stably expressing ULBP2 and evaluated the effects of ULBP2 on tumor progression using a syngeneic transplantation mouse model. We also investigated the impact of surface-expressed and soluble ULBP2 on NKG2D expression and the cytotoxic activity of murine splenocytes by performing in vitro assays. Our findings demonstrated that ULBP2 expressed on the surface of cancer cells promoted tumor growth by suppressing NK cell function, suggesting that modulating ULBP2-NKG2D interactions could be a potential target for cancer therapy.

## 2. Results

### 2.1. ULBP2 Suppresses Anti-Tumor Immunity via NKG2D and Promotes Tumor Growth

**Figure 1:**
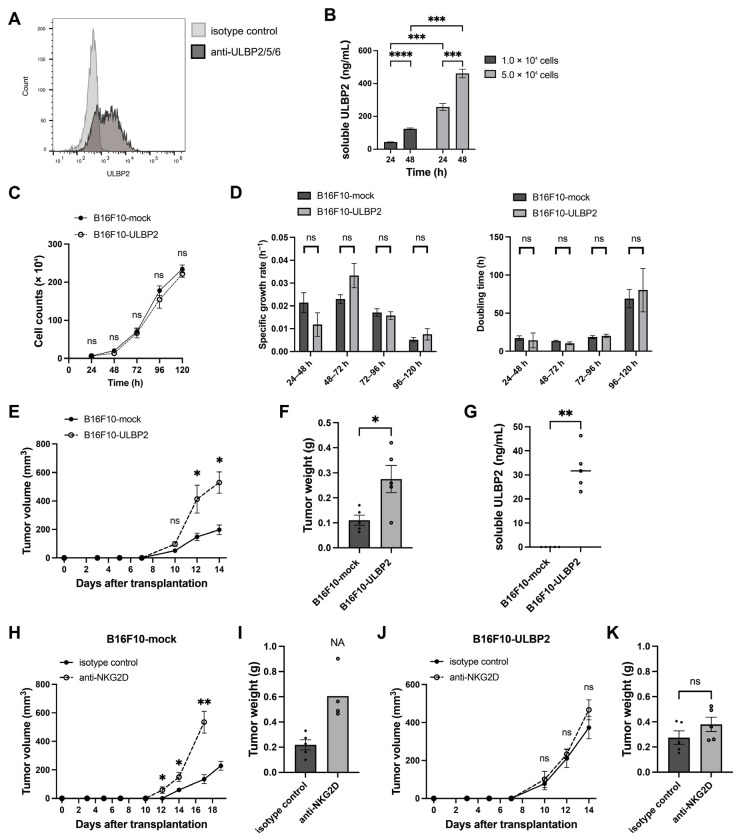
ULBP2 suppresses anti-tumor immunity via NKG2D and promotes tumor growth. (**A**) Flow cytometric evaluation of ULBP2 expression using B16F10-ULBP2 cells. The black and gray histograms represent staining with PE-conjugated anti-ULBP2/5/6 antibody and PE-conjugated isotype control antibody, respectively. (**B**) Measurement of soluble ULBP2 concentrations in the culture supernatants of B16F10-ULBP2 cells. Cells (1.0 × 10^4^ or 5.0 × 10^4^) were seeded in 24-well plates and cultured. Supernatants were collected at 24 and 48 h, and soluble ULBP2 levels were quantified using ELISA (*n* = 5). (**C**) Cell proliferation curves were generated by seeding cells into 6-well plates (5.0 × 10^4^ cells/well). Cells were harvested and counted every 24 h using a hemocytometer for 5 days (*n* = 6). (**D**) Specific growth rates (*μ*) and doubling times (*Td*) of cells calculated from the proliferation data in (**C**) using the formulas: *μ* = (ln *N*_t_ − ln *N*_0_) /*t* and *Td* = ln (2) /*μ* at 24-h intervals starting 24 h after seeding (*n* = 6). (**E**) Tumor growth curves of syngeneic subcutaneous tumors in C57BL/6 mice (*n* = 5) injected with B16F10-mock or B16F10-ULBP2 cells (1.0 × 10^6^). Tumor volumes were calculated as *V* = (width^2^ × length)/2. (**F**) Tumor weights were measured on day 14 for the mice in (**E**). (**G**) Soluble ULBP2 concentrations in plasma collected on day 14, measured using ELISA from the mice in (**E**). (**H**) Tumor growth in mice injected with B16F10-mock cells (3 × 10^5^) and treated with isotype control or anti-NKG2D antibody (200 μg/mouse) administered intraperitoneally twice per week starting on day 2 post-transplantation (*n* = 5). Due to the euthanization of one mouse because of tumor ulceration, the data point for day 17 post-transplantation in the anti-NK1.1 group was unavailable. (**I**) Tumor weights on day 19, post-transplantation, for the mice in (**H**). Statistical comparison was not performed due to data loss in the anti-NKG2D antibody group. NA indicates exclusion from statistical analysis. (**J**) Tumor growth in mice injected with B16F10-ULBP2 cells (3 × 10^5^) and treated as described in (**H**). (**K**) Tumor weights on day 14 for the mice in (**J**). In (**B**–**E**,**H**,**J**), data are presented as the mean ± standard error of the mean (SEM). * *p* < 0.05; ** *p* < 0.01; *** *p* < 0.001; **** *p* < 0.0001; ns: Not significant (two-tailed Welch’s *t*-test for (**B**); Mann–Whitney U test for (**C**–**E**,**H**,**J**)). In (**F**,**G**,**I**,**K**), individual values are shown with the mean ± SEM. * *p* < 0.05; ** *p* < 0.01; ns: Not significant (Mann–Whitney U test for (**F**,**G**,**K**)).

To evaluate the role of ULBP2 in anti-tumor immunity and tumor progression, we generated B16F10 murine melanoma cells stably expressing ULBP2 (B16F10-ULBP2). Surface expression of ULBP2 was confirmed via flow cytometry (Figure 1A), and soluble ULBP2 levels in the culture supernatants were measured using ELISA; we observed a cell density- and time-dependent increase in soluble ULBP2 (Figure 1B). Comparison of in vitro proliferation between B16F10-mock (control) and B16F10-ULBP2 showed no significant differences in the growth rate or doubling time (Figure 1C,D). However, when these cells were subcutaneously transplanted into syngeneic C57BL/6 mice, tumors derived from B16F10-ULBP2 grew faster than those from B16F10-mock (Figure 1E). Tumors were harvested on day 14, post-transplantation (Figure S1A). The tumor weight was significantly higher in the B16F10-ULBP2 group than in the B16F10-mock group (Figure 1F). Notably, soluble ULBP2 was detected only in the plasma obtained from mice bearing B16F10-ULBP2 tumors (Figure 1G).

To confirm that the rapid growth of B16F10-ULBP2 tumors was mediated by ULBP2-NKG2D interactions, we blocked NKG2D signaling by intraperitoneal administration of anti-NKG2D antibody (clone HMG2D). NKG2D blockade accelerated tumor growth in mice harboring B16F10-mock-derived tumors (Figure 1H). One mouse subjected to NKG2D blockade died on day 19, post-transplantation, due to peritoneal dissemination. Tumors from the remaining mice were harvested on the same day for analysis (Figure S1B and Figure 1I). In contrast, tumor growth in the B16F10-ULBP2 group was unaffected by NKG2D blockade (Figure 1J). All tumors were harvested on day 14, post-transplantation (Figure S1C), with no significant difference in tumor weight between the two groups (Figure 1K). These results suggest that NKG2D-mediated anti-tumor immunity suppresses the tumor growth in B16F10-mock tumors but is ineffective against B16F10-ULBP2 tumors.

### 2.2. Soluble ULBP2 Does Not Promote Tumor Growth

**Figure 2:**
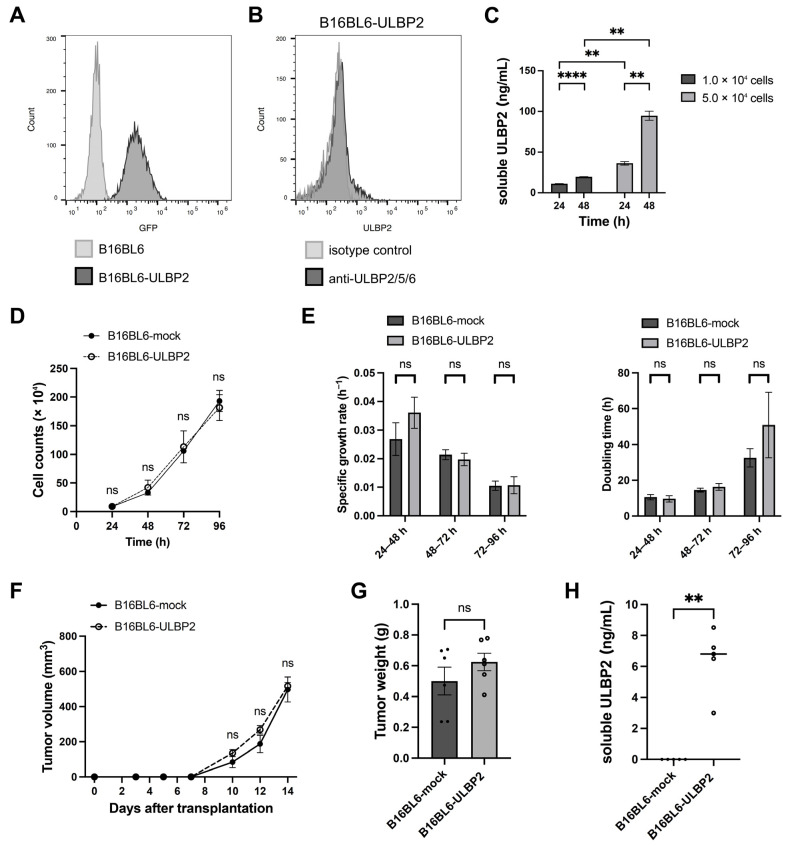
Soluble ULBP2 does not promote tumor growth. (**A**) Flow cytometric evaluation of GFP expression in B16BL6-ULBP2 cells. Black and gray histograms represent unstained B16BL6-ULBP2 cells and unstained wild-type B16BL6 cells, respectively. (**B**) Flow cytometric evaluation of ULBP2 expression in B16BL6-ULBP2 cells. Black and gray histograms represent cells stained with PE-conjugated anti-ULBP2/5/6 antibody and PE-conjugated isotype control antibody, respectively. (**C**) Measurement of soluble ULBP2 concentrations in the culture supernatants of B16BL6-ULBP2 cells. Cells (1.0 × 10^4^ or 5.0 × 10^4^) were seeded in 24-well plates and cultured. Supernatants were collected at 24 and 48 h, and soluble ULBP2 levels were quantified using ELISA (*n* = 3). (**D**) Cell proliferation curves of B16BL6-ULBP2 cells seeded in 6-well plates (5.0 × 10^4^ cells/well). Cells were harvested and counted every 24 h for 5 days using a hemocytometer (*n* = 6). (**E**) Specific growth rates (*μ*) and doubling times (*Td*) of cells calculated as described in (**D**). (**F**) Tumor growth curves of syngeneic subcutaneous tumors in C57BL/6 mice (*n* = 5) injected with B16BL6-mock or B16BL6-ULBP2 cells (1.0 × 10^6^). Tumor volumes were calculated as described in (**E**). (**G**) Tumor weights measured on day 14 for the mice in (**F**). (**H**) Soluble ULBP2 concentrations in plasma collected on day 14, measured using ELISA from the mice in (**F**). In (**C**–**F**), data are presented as the mean ± SEM. ** *p* < 0.01; **** *p* < 0.0001; ns: not significant (two-tailed Welch’s *t*-test for **C**; Mann–Whitney U test for (**D**–**F**)). In (**G,H**), individual values are shown with the mean ± SEM. ** *p* < 0.01; ns: not significant (Mann–Whitney U test).

As B16F10-ULBP2 cells suppressed anti-tumor immunity in a syngeneic subcutaneous transplantation mouse model, we next sought to determine whether surface-expressed or soluble ULBP2 was responsible for this effect. We aimed to generate B16BL6 cells stably expressing ULBP2; however, incidentally, all of the obtained clones lacked surface expression of ULBP2 and produced only soluble ULBP2. For subsequent experiments, one of these clones was selected and designated as B16BL6-ULBP2. Flow cytometry confirmed GFP expression but not surface ULBP2 expression (Figure 2A,B). Soluble ULBP2 in the culture supernatants increased in a cell density- and time-dependent manner (Figure 2C).

In vitro assays showed no significant differences between the proliferation of B16BL6-mock and B16BL6-ULBP2 cells (Figure 2D,E). Subcutaneous transplantation of these cells into syngeneic mice led to the formation of tumors with no significant differences in tumor growth rates between the groups (Figure 2F,G). Soluble ULBP2 levels in plasma were elevated in mice bearing B16BL6-ULBP2 tumors (Figure 2H). These results suggest that soluble ULBP2 alone does not influence tumor growth.

### 2.3. Surface-Expressed ULBP2 Downregulates NKG2D Expression on NK Cells

The distinction between surface-expressed and soluble ULBP2 in their effects on NKG2D expression next raised the question of how these forms of ULBP2 influence NKG2D expression. Prior research reported that co-culturing the NKG2D-expressing NKL cell line with C1R-ULBP2 cells led to a marked reduction in surface NKG2D expression, while supernatants from C1R-ULBP2 cells or control C1R-neo cells showed no such effect [16]. Building on these findings, we investigated the impact of surface-expressed and soluble ULBP2 on NKG2D expression in primary NK cells using B16F10-ULBP2 and B16BL6-ULBP2 cells.

**Figure 3:**
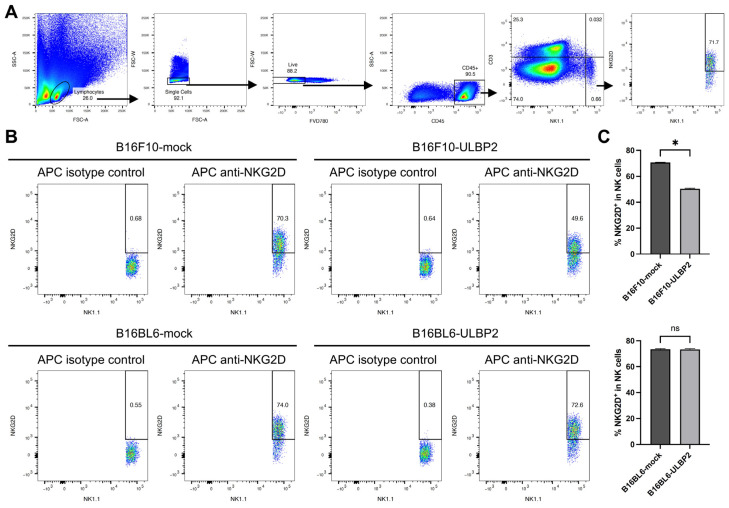
Surface-expressed ULBP2 downregulates NKG2D expression on NK cells. B16F10-mock, B16F10-ULBP2, B16BL6-mock, or B16BL6-ULBP2 cells (5.0 × 10^4^ cells) were seeded in 24-well flat-bottom plates and cultured for 24 h at 37 °C. Murine splenocytes (5.0 × 10^6^ cells) were then added to the culture along with IL-12 and IL-18 at a final concentration of 10 ng/mL each, and co-cultures were incubated for an additional 24 h. After incubation, splenocytes were harvested and analyzed by flow cytometry to measure the percentage of NKG2D^+^ cells among NK cells (CD45^+^CD3⁻NK1.1^+^ lymphocytes) (*n* = 4). (**A**) Gating strategy for flow cytometric analysis of splenocytes stained with the following fluorochrome-conjugated antibodies: BV605 anti-mouse CD45 (clone 30-F11), PerCP/Cy5.5 anti-mouse CD3 (clone 17A2), PE/Cy7 anti-mouse NK1.1 (clone PK136), and APC anti-mouse NKG2D (clone CX5). (**B**) Representative dot plots showing the gating for NKG2D^+^ NK cells. (**C**) Quantification of the percentage of NKG2D^+^ cells among NK cells. In (**C**), data are presented as the mean ± SEM. * *p* < 0.05; ns: not significant (Mann–Whitney U test).

Splenocytes from C57BL/6 mice were co-cultured for 24 h with B16F10-mock, B16F10-ULBP2, B16BL6-mock, or B16BL6-ULBP2 cells. NK cells were defined as CD45^+^CD3⁻NK1.1^+^ lymphocytes, and the percentage of NKG2D^+^ cells among NK cells was analyzed by flow cytometry (Figure 3A). Co-culture with B16F10-ULBP2 significantly reduced the percentage of NKG2D^+^ cells among NK cells compared to co-culture with B16F10-mock. In contrast, no such reduction was observed in co-cultures with B16BL6-ULBP2 compared to B16BL6-mock (Figure 3B,C). These results suggest that surface-expressed ULBP2, but not soluble ULBP2, downregulates NKG2D expression in NK cells.

### 2.4. ULBP2 Inhibits Anti-Tumor Immunity Mediated by NK Cells

**Figure 4:**
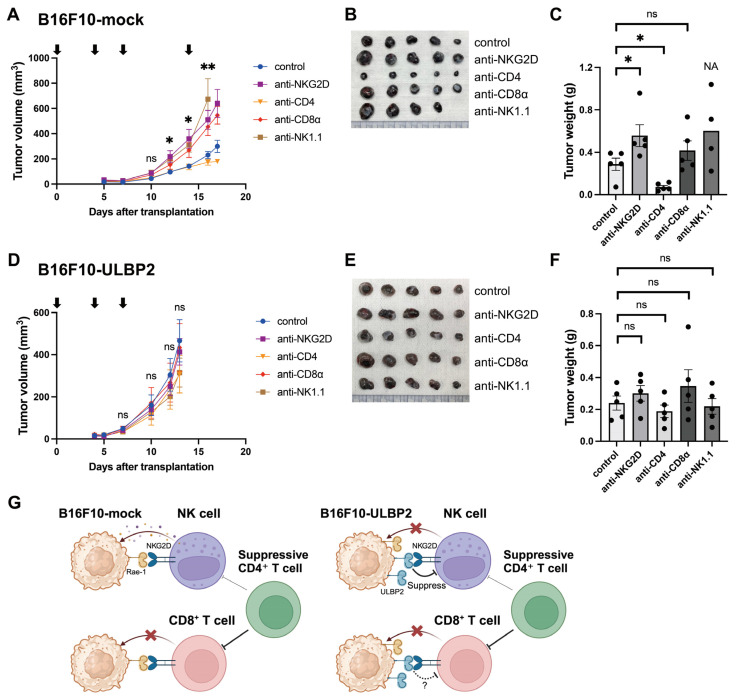
ULBP2 inhibits anti-tumor immunity mediated by NK cells. (**A**) Tumor growth in C57BL/6 mice subcutaneously transplanted with B16F10-mock cells (1 × 10^6^). Anti-NKG2D antibody (clone HMG2D), anti-mouse CD4 antibody (clone YTS191), anti-mouse CD8α antibody (clone 2.43), or anti-mouse NK1.1 antibody (clone PK136) was administered intraperitoneally at 300 μg/mouse on day 0 post-transplantation, followed by 200 μg/mouse on days 3, 7, and 13. PBS (−) was administered as a control on the same schedule. Arrows indicate treatment days. Tumor sizes were measured three times per week using an electronic caliper (*n* = 5). Due to the euthanization of one mouse because of tumor ulceration, the data point for day 17 post-transplantation in the anti-NK1.1 group was unavailable. (**B**) Photos of tumors harvested on day 17, post-transplantation, from the experiment shown in (**A**). (**C**) Tumor weights of all tumors harvested on day 17, post-transplantation, from (**A**). The anti-NK1.1 group was excluded from statistical comparisons due to data loss. NA indicates exclusion from statistical comparisons. (**D**) Tumor growth in C57BL/6 mice subcutaneously transplanted with B16F10-ULBP2 cells (1 × 10^6^) and treated as described in (**A**), except that no antibody was administered on day 13. Tumor growth was monitored as described above (*n* = 5). (**E**) Photos of tumors harvested on day 13 post-transplantation from the experiment shown in (**D**). (**F**) Tumor weights of all tumors harvested on day 13 post-transplantation from (**D**). (**G**) Schematic representation of the proposed mechanisms. A question mark and a dotted line indicate a potential mechanism suggested by our observations, but not directly demonstrated in this study. Illustration was created with BioRender.com. In (**A**,**D**), data are presented as the mean ± SEM. * *p* < 0.05; ** *p* < 0.01; ns: Not significant (Mann–Whitney U test: control vs. anti-NK1.1 group). In (**C**,**F**), individual values are shown with the mean ± SEM. * *p* < 0.05; ns: not significant (Mann–Whitney U test: control vs. each treatment group).

Building on the findings that B16F10-ULBP2 suppresses anti-tumor immunity via NKG2D in a syngeneic mouse model and on the in vitro observation that surface-expressed ULBP2 downregulates NKG2D expression on NK cells, we next sought to determine whether B16F10-ULBP2 suppresses NK cell-mediated anti-tumor immunity in vivo. Additionally, we investigated whether CD8^+^ or CD4^+^ T cells contributed to ULBP2-mediated tumor growth. To address these questions, C57BL/6 mice were treated with specific antibodies to deplete CD4^+^ T cells, CD8^+^ T cells, or NK cells, or to block NKG2D signaling. Phosphate-buffered saline (PBS) (−)-treated mice served as controls. B16F10-mock or B16F10-ULBP2 cells were subcutaneously transplanted into the right flank of these mice. In mice transplanted with B16F10-mock cells, tumors in the NK cell depletion group grew rapidly compared to those in the control group. However, one mouse was euthanized on day 17, post-transplantation, due to tumor ulceration, resulting in missing data for this group on day 17 (Figure 2A). Tumors from the remaining mice were harvested on day 17 for analysis. The tumor weights were significantly higher in the NKG2D blockade group and significantly lower in the CD4^+^ T cell depletion group compared to the control group (Figure 4B,C). In mice transplanted with B16F10-ULBP2 cells, all tumors were harvested on day 13 for analysis, and no significant differences in tumor weights were observed among the treatment groups (Figure 4D–F).

These findings suggest that NK cells suppress tumor growth in B16F10-mock tumors but are ineffective in suppressing the growth of B16F10-ULBP2 tumors. In contrast, CD4^+^ T cells promote tumor growth in B16F10-mock tumors but do not affect the growth of B16F10-ULBP2 tumors. This observation implies that suppressive CD4^+^ T cells, including Foxp3^+^CD25^+^ regulatory T cells (Tregs), and additional mechanisms suppress anti-tumor immunity in B16F10-ULBP2 tumors. A summary of these findings and proposed mechanisms is illustrated in Figure 4G.

### 2.5. Transient ULBP2 Exposure Enhances the Cytotoxic Activity of Splenocytes

**Figure 5:**
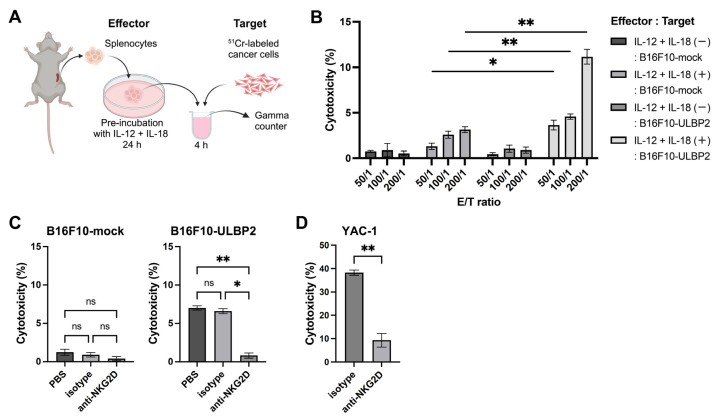
Transient ULBP2 exposure enhances the cytotoxic activity of splenocytes. (**A**) Schematic overview of the 4-h Chromium-51 (^51^Cr) release assay. ^51^Cr-labeled target cells were incubated with IL-12 (10 ng/mL) + IL-18 (10 ng/mL)-stimulated murine splenocytes (effector cells) for 4 h at 37 °C. After incubation, the supernatants were collected, and radioactivity was measured using a gamma counter. The illustration was created with BioRender.com. (**B**) Cytotoxic activity of murine splenocytes against B16F10-mock or B16F10-ULBP2 cells. Effector splenocytes were either stimulated with IL-12 (10 ng/mL) + IL-18 (10 ng/mL) or left unstimulated. Cytotoxicity was measured at effector-to-target (E/T) ratios of 50/1, 100/1, and 200/1. (**C**) NKG2D blockade experiments. Effector cells and ^51^Cr-labeled target cells (B16F10-mock or B16F10-ULBP2) were mixed at an E/T ratio of 200/1 and incubated with PBS (−), isotype control antibody (1 μg/mL), or anti-NKG2D antibody (clone HMG2D, 1 μg/mL) for 4 h. (**D**) Cytotoxic activity of splenocytes against ^51^Cr-labeled YAC-1 cells. Effector cells and target cells were mixed at an E/T ratio of 200/1 and incubated with isotype control antibody (1 μg/mL) or anti-NKG2D antibody (clone HMG2D, 1 μg/mL) for 4 h. In (**B**–**D**), data are presented as the mean ± SEM. * *p* < 0.05; ** *p* < 0.01; ns: not significant (Mann–Whitney U test for (**B**,**D**); Kruskal–Wallis test followed by Dunn’s multiple comparisons test for (**C**)).

The results described above demonstrated that surface-expressed ULBP2 suppresses NK cell-mediated anti-tumor immunity in vivo and downregulates NKG2D expression in vitro. To further investigate how ULBP2 influences NK cell function, we evaluated the cytotoxic activity of splenocytes using a 4-h Chromium-51 (^51^Cr) release assay [32,33]. Splenocytes isolated from C57BL/6 mice and pre-incubated with IL-12 and IL-18 for 24 h were used as effector cells. IL-12 and IL-18 are known to exert synergistic effects in promoting NK cell proliferation and activation [34]. The experimental workflow is illustrated in Figure 5A.

First, we compared cytotoxic activity under the conditions of with or without IL-12 and IL-18 stimulation during the pre-incubation of effector splenocytes, using either B16F10-mock or B16F10-ULBP2 as target cells at effector-to-target (E/T) ratios of 50/1, 100/1, and 200/1. Splenocytes stimulated with IL-12 and IL-18 exhibited cytotoxic activity against both B16F10-mock and B16F10-ULBP2 cells, with significantly higher activity observed against B16F10-ULBP2 at all E/T ratios (Figure 5B). This enhanced activity was significantly reduced by NKG2D blockade (Figure 5C). Similar reductions were observed against the YAC-1 target cells, which are sensitive to NK cells [35], upon NKG2D blockade (Figure 5D). These results suggest that transient ULBP2 exposure enhances cytotoxic activity via NKG2D.

It has been previously demonstrated that ULBP2 binds to murine NKG2D [14], and the present results confirm this function in murine immune cells, with its activation signals effectively blocked by the anti-mouse NKG2D antibody (clone HMG2D).

### 2.6. Sustained ULBP2 Exposure Suppresses the Cytotoxic Activity of Splenocytes

**Figure 6:**
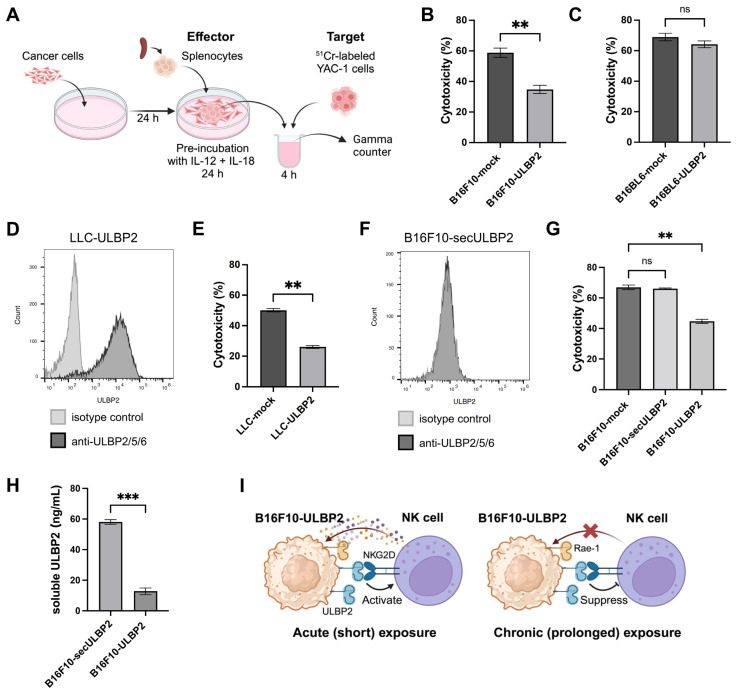
Sustained ULBP2 exposure attenuates the cytotoxic activity of splenocytes. (**A**) Schematic overview of the ^51^Cr release assay. Cancer cells (5.0 × 10^4^ for (**B**,**C**,**E**); 1.0 × 10^5^ for (**G**)) were seeded in 24-well flat-bottom plates and cultured for 24 h at 37 °C. Murine splenocytes (5.0 × 10^6^) were then added along with IL-12 and IL-18 (10 ng/mL each), and co-cultures were incubated for an additional 24 h. Splenocytes were subsequently harvested and used as effector cells in a ^51^Cr release assay, with ^51^Cr-labeled YAC-1 cells as targets at an effector-to-target (E/T) ratio of 200/1 (*n* = 6). The illustration was created with BioRender.com. (**B**) Cytotoxic activity of splenocytes co-cultured with B16F10-mock or B16F10-ULBP2 cells against YAC-1 cells. (**C**) Cytotoxic activity of splenocytes co-cultured with B16BL6-mock or B16BL6-ULBP2 cells against YAC-1 cells. (**D**) Flow cytometric analysis of ULBP2 expression in LLC-ULBP2 cells. Black and gray histograms represent staining with PE-conjugated anti-ULBP2/5/6 antibody and PE-conjugated isotype control antibody, respectively. (**E**) Cytotoxic activity of splenocytes co-cultured with LLC-mock or LLC-ULBP2 cells against YAC-1 cells. (**F**) Flow cytometric analysis of ULBP2 expression in B16F10-secULBP2 cells. Black and gray histograms represent staining with PE-conjugated anti-ULBP2/5/6 antibody and PE-conjugated isotype control antibody, respectively. (**G**) Cytotoxic activity of splenocytes co-cultured with B16F10-mock, B16F10-secULBP2, or B16F10-ULBP2 cells against YAC-1 cells. (**H**) Concentrations of soluble ULBP2 were measured in triplicate using representative culture supernatants selected from the co-culture experiments described in (**G**). (**I**) Illustration summarizing the effects of short- and long-term exposure to surface-expressed ULBP2 on NK cell cytotoxic activity (created with BioRender.com). In (**B**,**C**,**E**,**G**,**H)**, data are presented as the mean ± SEM. ** *p* < 0.01; *** *p* < 0.001; ns: not significant (Mann–Whitney U test for (**B**,**C**,**E**); Kruskal–Wallis test followed by Dunn’s multiple comparisons test for (**G**); two-tailed Welch’s *t*-test for (**H**)).

The observed enhancement of splenocyte cytotoxicity against B16F10-ULBP2 in transient interactions is consistent with previous reports on ULBP2-NKG2D interactions [6,16]. However, the rapid growth of B16F10-ULBP2 tumors in vivo suggests that prolonged exposure to ULBP2 in the tumor microenvironment may have an opposing effect. This observation aligns with previous reports that sustained stimulation of NKG2D by the murine ligand H60 impairs NKG2D functionality in NK cells, resulting in a reduction in their cytotoxic activity [36]. To investigate whether sustained exposure to ULBP2 similarly suppressed splenocyte cytotoxic activity, we co-cultured splenocytes from C57BL/6 mice with B16F10-mock or B16F10-ULBP2 cells for 24 h before using them as effector cells in a 4-h ^51^Cr release assay with YAC-1 cells used as targets (Figure 6A). Splenocytes co-cultured with B16F10-ULBP2 exhibited significantly reduced cytotoxic activity compared to those co-cultured with B16F10-mock (Figure 6B). In contrast, no significant differences were observed between splenocytes co-cultured with B16BL6-mock and B16BL6-ULBP2 cells, which lacked surface ULBP2 expression (Figure 6C). To further validate these findings, LLC-ULBP2 cells expressing surface ULBP2 were generated (Figure 6D). Splenocytes co-cultured with LLC-ULBP2 cells exhibited reduced cytotoxic activity compared to those co-cultured with LLC-mock (Figure 6E).

To confirm that this suppression was mediated by surface-expressed ULBP2, a B16F10-secULBP2 cell line was generated to produce soluble ULBP2 without its cell surface expression (Figure 6F). Splenocytes co-cultured with B16F10-secULBP2 showed no suppression of cytotoxic activity, whereas significant suppression was observed with splenocytes co-cultured with B16F10-ULBP2 (Figure 6G). Notably, soluble ULBP2 levels in the culture supernatants were higher in B16F10-secULBP2 co-cultures than in those with B16F10-ULBP2 (Figure 6H).

These findings demonstrate that sustained exposure to surface-expressed ULBP2 suppresses the cytotoxic activity of splenocytes in vitro. A schematic summary of these results is illustrated in Figure 6I.

## 3. Discussion

Recent comprehensive analyses of immune-related genes, using the Cancer Genome Atlas datasets and NanoString PanCancer Immune Profiling data, have identified ULBP2 as one of the top poor prognostic factors in both solid and hematopoietic malignancies [23,24,25,26,27,28,29,30,31]. These clinical findings suggest that ULBP2 expression on tumor cells suppresses anti-tumor immunity in a clinical context, thereby facilitating tumor progression. To elucidate the role of ULBP2 in immune evasion, we investigated its function in tumor cells. Using murine melanoma cell lines (B16F10 and B16BL6) engineered to stably express ULBP2, we found that these cells successfully engrafted into syngeneic C57BL/6 mice without causing immune rejection. Furthermore, we demonstrated that ULBP2 expressed on the tumor cell surface, suppressed NKG2D-mediated anti-tumor immunity, promoting tumor growth. To confirm that this effect was specifically mediated through the ULBP2-NKG2D interaction, we blocked this interaction with an anti-NKG2D antibody in subsequent experiments. As expected, administration of an anti-NKG2D antibody significantly promoted tumor growth in B16F10-mock tumors but did not further increase tumor growth in B16F10-ULBP2 tumors. In vitro experiments further revealed that this immunosuppressive effect was specifically mediated by surface-expressed ULBP2, as soluble ULBP2 did not exhibit similar activity. These findings underscore ULBP2 as a promising therapeutic target for enhancing anti-tumor immunity.

The observation that cell surface-expressed ULBP2, a ligand for the activating receptor NKG2D, suppresses anti-tumor immunity may initially seem contradictory to earlier reports [37]. The dual roles of NKG2D ligands in promoting tumor elimination and facilitating immune evasion have been extensively discussed [38]. For example, the ectopic expression of murine NKG2D ligands in tumor cells prevents tumor engraftment in syngeneic mice [9,10]. Conversely, soluble NKG2D ligands have been reported to downregulate NKG2D expression, thereby suppressing anti-tumor immunity [17,39]. Using bi-transgenic TRAMP mouse models of prostate cancer, soluble MICB was shown to promote carcinoma progression by depleting peripheral NK cells, whereas membrane-restricted MICB supported long-term tumor-free survival through sustained NKG2D-mediated anti-tumor immunity [40]. These findings have led to the emergence of therapeutic strategies that target NKG2D ligands by enhancing their cell surface expression and inhibiting their secretion [41].

However, the functions of NKG2D ligands are more complex. In a transgenic mouse model with skin-specific expression of Rae-1ε, sustained expression of NKG2D ligands led to NKG2D downregulation, increased incidence of skin cancer, and accelerated tumor progression [11]. Additionally, in vitro co-culture experiments demonstrated that chronic exposure to tumor cells expressing NKG2D ligands alters NKG2D signaling, enabling immune evasion [36,42]. These studies, which primarily investigated endogenous murine NKG2D ligands, did not focus on soluble ligands, but suggested that cell surface-expressed ligands can suppress anti-tumor immunity. Furthermore, a transgenic mouse model with systemic MICA expression reported suppression of NKG2D-mediated anti-tumor immunity, whereas soluble MICA in the serum did not affect NKG2D expression in non-transgenic mice [43]. This finding also supports the idea that cell surface expression is critical for immune suppression.

The fact that most human cancer cell lines derived from clinical specimens express NKG2D ligands has been attributed to their roles in stimulating inhibitory receptors on NK cells and suppressing anti-tumor immunity through soluble NKG2D ligands [38]. However, the potential for cell surface NKG2D ligands on cancer cells to suppress anti-tumor immunity and promote tumor growth in vivo remains insufficiently explored. Our findings suggest that targeting the persistent interaction between NKG2D and surface-expressed ULBP2 on cancer cells offers a promising therapeutic approach.

ULBP2, like MICA and MICB, is a human NKG2D ligand, and its ectopic expression in murine tumor cells carries the potential for immune rejection due to its xenogeneic nature. To address this issue, immunologically tolerant mouse models, such as MICAgen mice for MICA and MICB/B6 mice for MICB, have been developed [44,45]. These models provide valuable tools for studying xenogeneic NKG2D ligands while minimizing immune responses against xenogeneic antigens. In our experiments, we addressed the potential influence of immune responses against xenogeneic antigens by strictly confirming the equivalent proliferation capacity of control cells in vitro (Figure 1C,D) and conducting blocking experiments with an anti-NKG2D antibody, demonstrating that the observed effects were mediated by the ULBP2-NKG2D interaction. Moreover, results from CD8^+^ T cell and NK cell depletion experiments do not support the activation of cellular immunity against xenogeneic antigens in B16F10-ULBP2 tumors (Figure 4A–F).

Although NKG2D is a well-known activating receptor in NK cells, it also serves as a co-stimulatory molecule for T cells [12]. CD8^+^ T cells can kill tumor cells lacking MHC-I expression via NKG2D-NKG2D ligand interactions [46], highlighting that NKG2D and its ligands are potential targets for cancer immunotherapy. In the present study, CD8^+^ T cell depletion experiments did not provide direct evidence that ULBP2 suppresses CD8^+^ T cell-mediated anti-tumor immunity. Conversely, CD4^+^ T cell depletion reduced tumor growth in B16F10-mock tumors, consistent with the findings of a previous report [47]. Using B16F10 subcutaneous tumor models, their report demonstrated that administration of an anti-CD4 antibody had strong anti-tumor effects, which were completely reversed by CD8^+^ T cell depletion. Their findings also showed that treatment with an anti-CD25 antibody, which is widely used to deplete Foxp3^+^CD25^+^ Tregs in mice, failed to reduce tumor growth, likely due to the simultaneous depletion of tumor-specific CD8^+^ T cells [47]. Based on these findings, the tumor growth suppression observed in B16F10-mock tumors during CD4^+^ T cell depletion experiments may be primarily attributed to the removal of suppressive CD4^+^ T cells, including Foxp3^+^CD25^+^ Tregs, which may have enhanced CD8^+^ T cell-mediated anti-tumor immunity. Consistent with these findings, CD4-targeted approaches have been explored in cancer immunotherapy to enhance anti-tumor immunity [48]. However, the effectiveness of these approaches can vary depending on the tumor microenvironment and immune context [49]. In contrast, the lack of an effect of CD4^+^ T cell depletion on B16F10-ULBP2 tumor growth suggests that additional mechanisms, potentially via direct interference with NKG2D on CD8^+^ T cells, contribute to the suppression of anti-tumor immunity in this context (Figure 4G). Nevertheless, the potential contribution of NKG2D-expressing CD4^+^ T cells or helper T cell subpopulation depletion, or other tumor microenvironment factors to the suppression of CD8^+^ T cell function in B16F10-ULBP2 tumors cannot be ruled out and warrants further investigation. Additionally, B16F10 tumors exhibit inherently low CD8^+^ T cell infiltration, which classifies them as “cold” tumors [50]. This characteristic may limit the detectable effects of immune cell depletion in our model. Future studies should include “hot” tumors with robust immune infiltration to better understand how ULBP2 affects the tumor microenvironment.

One major limitation of this study is the use of murine cells ectopically expressing the human NKG2D ligand ULBP2. As mentioned earlier, we cannot rule out that immune responses against xenogeneic antigens influenced the results. Moreover, the functions of ectopically expressed ULBP2 may differ from those of endogenously expressed ULBP2 in human cancer cells. Specifically, soluble ULBP2 secreted by engineered cells may differ structurally and functionally from soluble ULBP2 derived from the cleavage of surface-expressed ULBP2. Furthermore, whether ULBP2 exerts the same immunosuppressive effects on human NKG2D-expressing cells as observed in this study remains uncertain. To address these limitations, future research employing tumor-bearing animal models with fully humanized immune systems will be essential. Another limitation lies in the use of a non-spontaneous tumor model. The findings may not fully reflect the role of ULBP2 in the early stages of tumorigenesis. It is possible that the results are more applicable to specific phases of cancer progression, potentially limited to later stages. Nevertheless, the study suggests that surface-expressed ULBP2 may not consistently influence tumor elimination across all stages of tumor development.

In conclusion, our study demonstrates that surface-expressed ULBP2, but not soluble ULBP2, plays a critical role in tumor progression by modulating NKG2D-mediated immune responses. These findings expand our understanding of the role of ULBP2 in tumor biology and provide a foundation for the development of targeted therapies to enhance anti-tumor immunity.

## 4. Materials and Methods

### 4.1. Cell Lines

B16F10, B16BL6, YAC-1, and LLC cell lines were purchased from RIKEN BioResource Research Center (Tsukuba, Japan). All cell lines were used before ten passages after obtaining them from the vendor. B16F10, B16BL6, and YAC-1 cells were cultured in RPMI-1640 medium containing 10% fetal bovine serum (FBS) and 1× penicillin-streptomycin (15140122, Thermo Fisher Scientific; Waltham, MA, USA) at 37 °C in a 5% CO_2_ atmosphere. LLC cells were cultured in DMEM containing 10% FBS and 1× penicillin-streptomycin at 37 °C in a 5% CO_2_ atmosphere. Cell lines were tested for *Mycoplasma* and have not been reauthenticated.

### 4.2. Cell Lines Stably Expressing ULBP2

Stable ULBP2-expressing cell lines, including B16F10-ULBP2, B16BL6-ULBP2, and LLC-ULBP2, were established as follows. The pcDNA3.1(+) IRES GFP vector, a gift from Kathleen_L Collins (Addgene plasmid # 51406; http://n2t.net/addgene:51406 (accessed on 1 March 2025); RRID:Addgene_51406) [51], was used as the backbone. ULBP2 cDNA (RefSeq: NM_025217.4) was inserted into the vector, and cells were transfected using Lipofectamine 3000 (Invitrogen, Carlsbad, CA, USA). After transfection, cells were selected using G418 (Sigma-Aldrich; St. Louis, MO, USA). To isolate ULBP2-expressing clones, cells were stained with a PE-conjugated anti-ULBP2/5/6 antibody (clone 165903, FAB1298P, R&D Systems; Minneapolis, MN, USA; RRID:AB_2214693). GFP- and PE-positive cells were subjected to single-cell sorting on a MoFlo XDP cell sorter (Beckman Coulter; Brea, CA, USA). If GFP- and PE-double-positive cells could not be obtained, single-cell sorting was performed for GFP-positive cells alone. After 1–2 weeks of culture, the concentration of soluble ULBP2 in the culture supernatants was measured using the Human ULBP2 ELISA Kit (DY1298, R&D Systems). ULBP2 expression on the cell surface was analyzed by performing flow cytometry following staining with the PE-conjugated anti-ULBP2/5/6 antibody. To generate B16F10-secULBP2 cell lines, the 1–651-bp region of ULBP2 cDNA (RefSeq: NM_025217.4) was inserted into the pcDNA3.1(+) IRES GFP vector, and stable cell lines were established using the methods described above. Control cell lines (B16F10-mock, B16BL6-mock, and LLC-mock) were generated by transfecting cells with an empty pcDNA3.1(+) IRES GFP vector, followed by selection with G418 under the same conditions.

### 4.3. ELISA

Concentrations of soluble ULBP2 in the culture supernatants and plasma collected from mice were measured using the Human ULBP2 ELISA kit (DY1298, R&D Systems), according to the manufacturer’s instructions. Plasma samples from mice were diluted 1:3 with the Reagent Diluent (DY995, R&D Systems), and the culture supernatants were diluted 1:50 before being assayed. 

### 4.4. In Vitro Proliferation Assay

Cell proliferation curves were generated by seeding cells into 6-well plates (5.0 × 10^4^ cells/well). Cells were counted every 24 h using a hemocytometer for 4–5 days. The specific growth rate (*μ*) was calculated using the formula: *μ* = (ln *N*_t_ − ln *N*_0_) /*t*, where *N*_t_ and *N*_0_ represent the cell numbers at the final and initial time points, respectively. The cell doubling time (*T_d_*) was calculated using the formula: *T_d_* = ln (2) /*μ*. The specific growth rate (*μ*) and cell doubling time (*T_d_*) were calculated at 24-h intervals starting 24 h after seeding.

### 4.5. Flow Cytometry for Cancer Cell Lines

Cells were harvested and resuspended in PBS (−) (164-23551, Fujifilm Wako Chemicals; Osaka, Japan) containing 0.5% bovine serum albumin (BSA). Cells were then stained with PE-conjugated anti-ULBP2/5/6 antibody. Flow cytometry was performed using either an LSRFortessa^TM^ X-20 (BD Biosciences; Franklin Lakes, NJ, USA) or a CytoFLEX S (Beckman Coulter). Data were analyzed using FlowJo^TM^ software, version 10.10 (BD Biosciences).

### 4.6. Mice

C57BL/6JJcl mice (RRID:IMSR_JCL:JCL:MIN-0003) were purchased from CLEA Japan, Inc. (Tokyo, Japan) and maintained under specific pathogen-free conditions; females aged 6 weeks were used in the experiments.

### 4.7. Co-Culture of Cancer Cell Lines and Murine Splenocytes

Cancer cell lines (B16F10-mock, B16F10-ULBP2, B16BL6-mock, B16BL6-ULBP2, LLC-mock, LLC-ULBP2, or B16F10-secULBP2) (5.0 × 10^4^ or 1.0 × 10^5^ cells) were seeded in 24-well flat-bottom plates and cultured in RPMI-1640 medium supplemented with 10% FBS and 1× penicillin-streptomycin for 24 h at 37 °C in a 5% CO_2_ atmosphere. Splenocytes isolated from 6-week-old female C57BL/6 mice (5.0 × 10^6^ cells) were then added to the culture along with recombinant mouse IL-12 (p70) (577004, BioLegend; San Diego, CA, USA) and recombinant mouse IL-18 (767004, BioLegend) at a final concentration of 10 ng/mL each. The co-culture was incubated for an additional 24 h under the same conditions. After incubation, murine splenocytes were harvested and subjected to flow cytometric analysis or a 4-h Chromium-51 (^51^Cr) release assay.

### 4.8. Flow Cytometric Analysis for Murine Splenic NK Cells

Murine splenocytes were collected, resuspended in PBS (−) containing 0.5% BSA and 2 mM EDTA and stained with eBioscience™ Fixable Viability Dye eFluor™ 780 (65-0865-14, Thermo Fisher Scientific) to assess viability. Fc receptors were blocked using TruStain FcX™ PLUS (anti-mouse CD16/32) Antibody (clone S17011E, 156604, BioLegend; RRID:AB_2783138). After blocking, cells were incubated with the following fluorochrome-conjugated antibodies for surface marker analysis: BV605 anti-mouse CD45 (clone 30-F11, 103140, BioLegend; RRID:AB_2562342), PerCP/Cy5.5 anti-mouse CD3 (clone 17A2 , 100218, BioLegend; RRID:AB_1595492), PE/Cy7 anti-mouse NK1.1 (clone PK136, 108714, BioLegend; RRID:AB_389364), and APC anti-mouse NKG2D (clone CX5, 130212, BioLegend; RRID:AB_1236372). Samples were acquired on an LSRFortessa^TM^ X-20 (BD Biosciences). Data were analyzed using FlowJo^TM^ software, version 10.10 (BD Biosciences).

### 4.9. Tumor Transplantation and In Vivo Experimental Procedures

Upon arrival at the experimental facility, C57BL/6 mice were randomly assigned to groups of 5–6 animals per cage and acclimatized for one week to minimize stress and allow for adaptation to the environment. After the acclimatization period, B16F10-mock, B16F10-ULBP2, B16BL6-mock, or B16BL6-ULBP2 cells (3 × 10^5^ or 1 × 10^6^ cells) were suspended in 100 μL of PBS (−) and subcutaneously transplanted into the right flank of 6-week-old female C57BL/6 mice. Tumor sizes were measured three times per week using an electronic caliper, and tumor volumes were calculated using the formula (length × width^2^) /2. The humane endpoint criteria resulting in euthanasia or termination of the experiment were defined as follows: the tumor weight, estimated from the calculated tumor volume after subcutaneous injection, exceeded 10% of the animal’s body weight; the tumor exhibited ulceration, necrosis, or infection; or the animal’s body weight decreased by more than 20% compared with control animals. The experimental endpoint was defined as tumor volume and tumor weight, which were measured when any mouse within a given tumor cell transplant group had reached or was expected to reach the humane endpoint by the next measurement. At this point, all mice transplanted with the same tumor cells were sacrificed, and their tumors were excised for weight comparison among experimental groups. A schematic representation of the experimental design is provided in Figure S2.

NKG2D blockade, CD4^+^ T cell depletion, CD8^+^ T cell depletion, and NK cell depletion were performed by intraperitoneally administering anti-mouse NKG2D (clone HMG2D, BE0111, Bio X Cell; Lebanon, NH, USA; RRID:AB_10950118), anti-mouse CD4 (clone YTS191, BE0119, Bio X Cell; RRID:AB_10950382), anti-mouse CD8α (clone 2.43, BE0061, Bio X Cell; RRID:AB_1125541), and anti-mouse NK1.1 antibodies (clone PK136, BE0036, Bio X Cell; RRID:AB_1107737). As controls, we used either polyclonal Armenian hamster IgG (BE0091, Bio X Cell, RRID:AB_1107773) or PBS (−). The dosage and administration schedule are described in the figure legends.

### 4.10. Four-Hour ^51^Cr Release Assay

The 4-h ^51^Cr release assay was performed as described previously [52]. Briefly, target cells were incubated with 100 μCi ^51^Cr (Perkin-Elmer Life and Analytical Sciences, Boston, MA, USA) for 1 h. After labeling, cells (10^4^ per well) were washed and co-incubated with effector cells at E/T ratios of 50/1, 100/1, or 200/1 in a 96-well U-bottom plate for 4 h. Both the ^51^Cr labeling and the co-culture were performed in RPMI-1640 medium supplemented with 10% FBS and 1× penicillin-streptomycin at 37 °C in a humidified atmosphere containing 5% CO_2_. Following incubation, the supernatant was collected, and the radioactivity of the released ^51^Cr was measured using a gamma counter. The percentage of specific lysis was calculated using the formula: percentage specific lysis = [(counts per min of effector cells − spontaneous counts per min)/(total counts per min − spontaneous counts per min)] × 100.

### 4.11. Statistical Analysis

Statistical analyses were performed using GraphPad Prism version 10. For the comparison of sULBP2 concentrations in culture supernatants, normality was confirmed using the Shapiro-Wilk test, and the two-tailed Welch’s *t*-test was performed. For other comparisons between the two groups, the Mann–Whitney U test was used, whereas differences among multiple groups were analyzed using the Kruskal–Wallis test. Data are presented as individual values or means, with error bars representing the standard error of the mean. Statistical significance was defined as *p* < 0.05.

## Data Availability

The original contributions presented in this study are included in the article/Supplementary Materials. Further inquiries can be directed to the corresponding author.

## Supplementary Materials

The following supporting information can be downloaded at: https://www.mdpi.com/article/10.3390/ijms26072950/s1.
