# Supplementary material for: ULBP2 Promotes Tumor Progression by Suppressing NKG2D-Mediated Anti-Tumor Immunity
Source: Int J Mol Sci. 2025 Mar 24;26(7):2950. doi: 10.3390/ijms26072950 (PMC11988498; doi:10.3390/ijms26072950)
Supplement: Supplementary file 1 [file ijms-26-02950-s001.zip › ijms-3466732-supplementary.pdf]

Supplementary Materials

ULBP2 Promotes Tumor Progression by Suppressing NKG2D-Mediated Antitumor Immunity

Kohei Yamane, Kosuke Yamaguchi, Yasuhiko Teruya, Naomi Miyake, Yuji Nakayama, Takafumi Nonaka, Hiroki Chikumi, Akira Yamasaki

This Supplementary Materials file includes two supplementary figures.

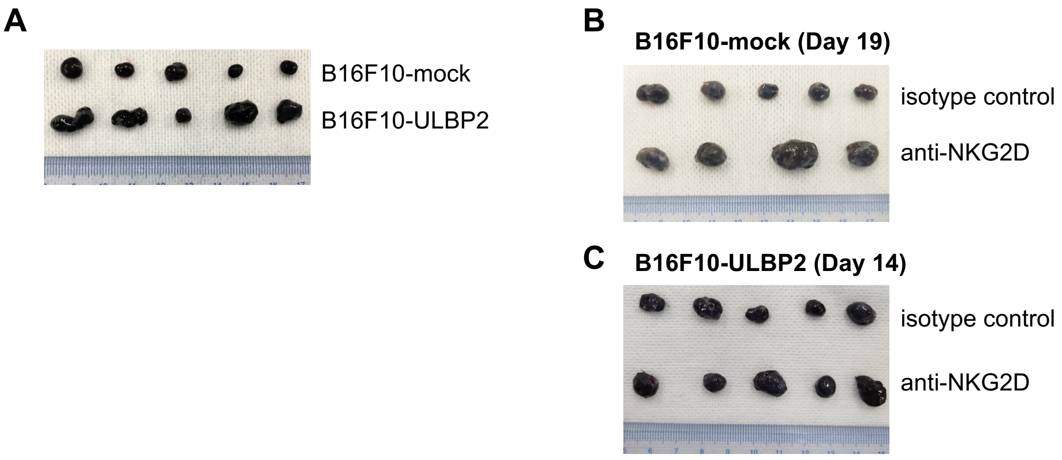

Figure S1. Supplementary data for **Figure 1**. Photos of tumors corresponding to the tumor weights shown in the graphs of **Figure 1F** (**A**)**, Figure 1I** (**B**)**,** and **Figure 1K** (**C**)**.**

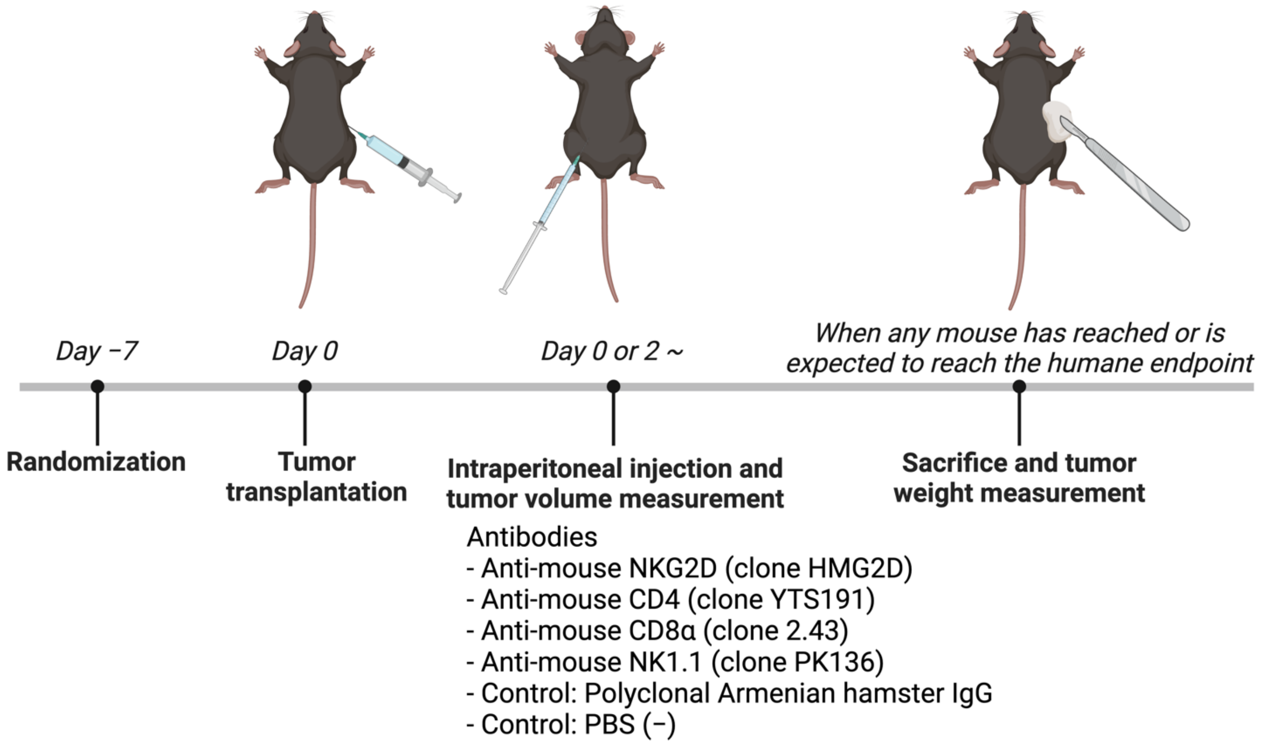

Figure S2. Schematic representation of the tumor transplantation experiments. C57BL/6 mice were randomly assigned to groups and acclimatized for one week before tumor transplantation (Day −7). On Day 0, B16F10-mock, B16F10-ULBP2, B16BL6-mock, or B16BL6-ULBP2 cells were subcutaneously injected into the right flank. Intraperitoneal injections of antibodies or control treatments were initiated on Day 0 or Day 2, according to the experimental schedule, along with tumor volume measurements. When any mouse within a given tumor cell transplant group had reached or was expected to reach the humane endpoint by the next measurement, all mice transplanted with the same tumor cells were sacrificed, and tumor weights were measured for comparison among experimental groups. Created with BioRender.com.
